# The Role of Artificial Intelligence in Prognosis, Recurrence Prediction, and Treatment Outcomes in Laryngeal Cancer: A Systematic Review

**DOI:** 10.3390/cancers18081257

**Published:** 2026-04-16

**Authors:** Hadi Afandi Al-Hakami, Ismail A. Abdullah, Nora S. Almutairi, Rimaz R. Aldawsari, Ghadah Ali Alluqmani, Halah Ahmed Fallatah, Yara Saud Alsulami, Elyas Mohammed Alasiri, Rahaf D. Alsufyani, Raghad Ayman Alorabi, Reffal Mohammad Aldainiy

**Affiliations:** 1 Otolaryngology/Head and Neck Section, Department of Surgery, Ministry of the National Guard-Health Affairs, Jeddah 22384, Saudi Arabia; hakamiha@mngha.med.sa; 2 College of Medicine, King Saud bin Abdulaziz University for Health Sciences, Jeddah 22384, Saudi Arabia; halahfallatah0@gmail.com (H.A.F.); yarasaud18@gmail.com (Y.S.A.); 421220069@ksau-hs.edu.sa (R.A.A.); reffal.aldainiy@gmail.com (R.M.A.); 3 King Abdullah International Medical Research Center, Jeddah 22384, Saudi Arabia; iaabdullah@alfaisal.edu (I.A.A.); nmma80@hotmail.com (N.S.A.); remazrashed1@gmail.com (R.R.A.); ye200010@gmail.com (E.M.A.); dr.rahafd@gmail.com (R.D.A.); 4 College of Medicine, Alfaisal University, Riyadh 11533, Saudi Arabia; 5 College of Medicine, King Saud bin Abdulaziz University for Health Sciences, Riyadh 11481, Saudi Arabia; 6 College of Medicine, University of Jeddah, Jeddah 21589, Saudi Arabia; 7 College of Medicine, Albaha University, Albaha 65779, Saudi Arabia; 8 College of Medicine, Taif University, Taif 21974, Saudi Arabia

**Keywords:** laryngeal cancer, artificial intelligence, deep learning, prognosis, treatment outcomes, recurrence

## Abstract

**Background:** Laryngeal cancer (LC), a common subtype of head and neck cancers (HNC), is most frequently represented by laryngeal squamous cell carcinoma (LSCC). Prognosis largely depends on early detection; however, traditional prognostic tools, including tumor-node-metastasis (TNM) staging, often show limited predictive accuracy. Artificial intelligence (AI), including machine learning (ML), natural language processing, and deep learning (DL), has emerged as a promising approach to improving cancer diagnosis, prognosis, and treatment planning by analyzing clinical data and medical imaging. **Objective:** This systematic review assesses the role of AI in prognosis, recurrence prediction, and treatment outcomes in LC. **Methods:** PubMed, MEDLINE, Scopus, Web of Science, IEEE Xplore, and ScienceDirect were searched up to January 2025. A total of 1062 records were identified; after title/abstract screening and full-text assessment, 29 studies were included. Eligible studies involved adult patients with LC and applied AI to diagnose, prognose, predict recurrence, or assess treatment outcomes using human datasets. Study quality and risk of bias were evaluated using the QUADAS-2 and QUIPS. **Results:** The 29 included studies were mostly retrospective, with sample sizes ranging from 10 to 63,000 patients. Most focused on LSCC, with a higher prevalence in males. The studies utilized various AI techniques, including deep learning models such as convolutional neural networks (CNNs) and DeepSurv, as well as ML algorithms like random survival forest, gradient boosting machines, random forest, k-nearest neighbors, naïve Bayes, and decision trees. AI models demonstrated strong prognostic performance, surpassing Cox regression and TNM staging in predicting survival and recurrence. Several studies reported outcomes related to treatment, such as chemotherapy response, occult lymph node metastasis, and the need for salvage surgery. Methodological quality varied, with biases related to patient selection and confounding factors. **Conclusions:** AI has the potential to improve prognosis estimation, recurrence prediction, and treatment outcome assessment in LC. However, although AI can be a helpful addition to clinical decision-making, more prospective studies, external validation, and standardized evaluation are necessary before these technologies can be confidently adopted in everyday clinical practice.

## Simple Summary

Predicting response to treatment and prognosis remains a challenging aspect in laryngeal cancer since traditional methods, such as the tumor staging system, do not always reflect the complexity of the disease. Recent advances in artificial intelligence have provided high potential in analyzing complex medical data and improving predictions in cancer care. This study was conducted to systematically evaluate how artificial intelligence has been used to predict prognosis, recurrence, and treatment outcomes in laryngeal cancer, and to assess the quality and reliability of these approaches. By summarizing current evidence, this review aims to inform researchers and clinicians about the potential benefits and limitations of AI-based models, support the development of more accurate prognostic tools, and guide future research toward standardized, interpretable, and clinically applicable AI systems in head and neck oncology.

## 1. Introduction

Head and Neck Cancer (HNC) refers to various malignant tumors that develop around the pharynx, larynx, paranasal sinuses, or oral cavity. Over the past few decades, the incidence of HNC has increased, with lower survival rates reflecting the disease’s impact on the population [1]. Laryngeal Cancer (LC), a malignant tumor originating from the mucous membrane of the larynx, is one of the more common types of HNC, representing up to one-third of cases [2,3,4]. Recent studies indicate a trend of younger individuals being diagnosed with LC, primarily due to tobacco use; other risk factors include alcohol consumption, HPV and EBV infections, and opium use [5,6]. LSCC is the most common type of LC, originating from the stratified squamous epithelium lining the larynx. Its development begins with epithelial hyperplasia and dysplasia, progressing to carcinoma in situ and eventually invasive carcinoma. Histologically, these tumors display malignant squamous cells featuring keratinization, intercellular bridges, nuclear atypia, and increased mitotic activity. Molecular changes, including mutations in tumor suppressor genes and overexpression of epidermal growth factor receptor (EGFR), play significant roles in laryngeal carcinogenesis [6,7]. Diagnosing LC begins with a comprehensive history and physical examination; this is followed by tissue acquisition through various methods, such as biopsy during direct laryngoscopy for suspected lesions or fine-needle aspiration cytology for suspected nodal lesions [7]. The prognosis for LC depends on the stage of the disease at the time of diagnosis and treatment. In other words, diagnosing late-stage disease is ultimately linked to a poorer prognosis and outcome, while early-stage detection can increase survival rates to 80% [7].

Despite advances in imaging and pathological analysis, current prognostic tools, such as TNM staging and traditional models, for LC remain limited in accuracy. A study by Hoban et al. highlighted these shortcomings, showing that four commonly used prognostic calculators significantly underestimated survival rates in patients with LC. The predicted 5-year survival was 47.7%, but the actual observed rate was 71.9%. Additionally, the models demonstrated only moderate discriminative power (C-indexes of 0.66–0.68). These limitations emphasize the need for more precise and personalized prognostic models and AI-driven decision-making tools [8]. Examples of such models include ML, natural language processing (NLP), and deep learning (DL) [9].

AI has the tremendous ability to revolutionize and improve healthcare to new heights. ML algorithms are a type of AI that learns from data, such as electronic medical records (EMR), and uses this information to make accurate predictions and better decisions with minimal human guidance. This enhances performance in clinical scenarios [9]. NLP, another branch of AI, is used to bridge the gap between human communication and computer understanding. It is a useful tool that can extract vital information from unstructured text, such as EMRs, doctors’ notes, and medical research, which can then be applied in decision-making [9]. Likewise, DL models utilize structures called “neural networks,” which have been used in image analysis such as X-rays, Magnetic Resonance Imaging (MRIs), and Computed Tomography (CT) scans. They are trained on numerous images of normal and cancerous lesions to distinguish between them, identify patterns, and assist in diagnosis and recurrence detection [9].

Currently, AI is an emerging technology in many fields, but specifically in oncology, DL models have accurately diagnosed cancer and its subtypes directly from histopathological and other medical images [10,11]. Using radiomics, which converts medical images into quantitative high-dimensional data, AI has been proven to improve early detection, staging, and prognosis more accurately [12]. In contrast to traditional biopsies, which require general anaesthesia, radiomics enables comprehensive analysis of the entire tumor without anaesthesia, offering a complete understanding of tumor characteristics and progression [13]. This tool can potentially lead to more effective and cost-efficient care for patients with HNC. Furthermore, AI can integrate clinical data to aid personalized treatment and real-time decision-making in the operating room, potentially reducing the risk of complications [14].

Despite its revolutionary promises, several limitations should be considered. Integrating AI into a developed healthcare system requires significant changes, including establishing standardized guidelines, staff training, and ensuring that data is incorporated without bias [15]. Another limitation is that AI operates as a “black-box model,” which can lead to challenges among physicians as they try to uncover how it arrived at a particular conclusion [15].

This study aims to conduct a systematic review on the role of AI in prognosis, recurrence predictions, and treatment outcomes in LC. It will seek to evaluate the effectiveness of AI and its ability to preserve organ function and reduce mortality rates.

## 2. Methods

A systematic review of the literature was conducted in accordance with the Preferred Reporting Items for Systematic Reviews and Meta-Analyses (PRISMA) guidelines [16], and the protocol was registered in PROSPERO under the number CRD420261290303. Institutional Review Board approval was obtained from King Abdullah International Medical Research Center, Jeddah, Saudi Arabia (approval number NRJ25/028/12). This review attempted to synthesize evidence regarding how AI can be used to predict prognoses, recurrences, and measure treatment outcomes for patients with LC.

### 2.1. Eligibility Criteria

We reviewed all clinical studies involving adults with LC, mainly LSCC, spanning all anatomical subsites (glottic, supraglottic, and subglottic) and clinical stages (I–IV), where AI techniques were applied to diagnostic classification, prognosis prediction, recurrence prediction, or treatment outcome. Studies were required to utilize human-derived datasets such as clinical records, imaging modalities including computed tomography (CT), magnetic resonance imaging (MRI), positron emission tomography (PET), laryngoscopy, narrow-band imaging, or hyperspectral imaging, as well as radiomic or transcriptomic datasets such as Gene Expression Omnibus (GEO) or The Cancer Genome Atlas (TCGA). Included studies were required to report quantitative performance metrics evaluating the predictive performance of AI models. These metrics included measures such as area under the receiver operating characteristic curve (AUC-ROC), sensitivity, specificity, accuracy, precision, positive predictive value, negative predictive value, or survival-related prognostic metrics such as hazard ratios or concordance indices. For this review, adequate validation of artificial intelligence (AI) models was defined as the implementation of a recognized validation strategy demonstrating model performance beyond the training dataset. Acceptable validation approaches included internal validation methods such as k-fold cross-validation, bootstrapping, or hold-out validation using an independent testing subset of the dataset, as well as external validation using an independent dataset obtained from a different patient cohort or institution. Studies reporting only training performance without any form of validation or an independent testing dataset were considered methodologically insufficient and were excluded. Studies were excluded if they did not employ AI-based analytical approaches, focused exclusively on algorithm development without clinical application, lacked clear reporting of model performance metrics, or were non-original publications such as narrative reviews, systematic reviews, editorials, commentaries, letters, or opinion pieces. Studies involving animal models, purely simulated datasets without clinical patient data, or studies lacking a defined validation strategy were also excluded.

### 2.2. Literature Search Strategy

A comprehensive literature search was conducted across multiple electronic databases, including PubMed, MEDLINE, Scopus, Web of Science, IEEE Xplore, and ScienceDirect. The search covered all records from database inception until January 2025. The search strategy aimed to identify studies evaluating the use of AI methods for diagnosis, prognosis prediction, recurrence prediction, or treatment outcome prediction in LC. The search strategy combined Medical Subject Headings (MeSH) terms and free-text keywords related to LC and AI methodologies. The core search strategy applied in PubMed was structured as follows: (“laryngeal cancer” OR “laryngeal carcinoma” OR “laryngeal squamous cell carcinoma” OR “LSCC”) AND (“artificial intelligence” OR “machine learning” OR “deep learning” OR “radiomics” OR “neural network*” OR “convolutional neural network*” OR “support vector machine” OR “random forest”) AND (“prognosis” OR “prognostic prediction” OR “recurrence” OR “recurrence prediction” OR “treatment outcome*” OR “survival prediction” OR “treatment response”). Equivalent search strategies adapted to the indexing systems of each database were applied for MEDLINE, Scopus, Web of Science, IEEE Xplore, and ScienceDirect. Searches were restricted to studies published in English. To ensure completeness, the reference lists of all included studies and relevant review articles were manually screened to identify additional studies that may not have been captured through the electronic database search.

### 2.3. Selection of Articles and Data Extraction

**Figure 1:**
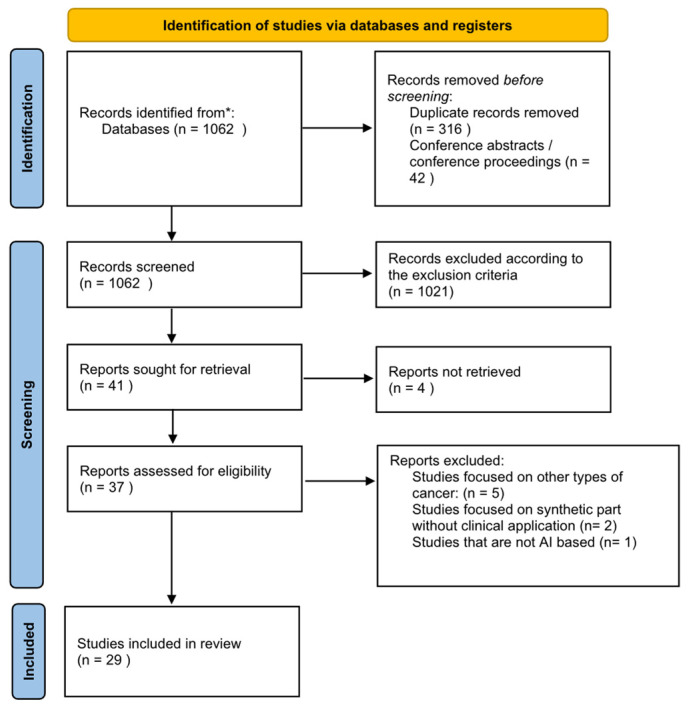
PRISMA flow chart. *: Databases include PubMed, Medline, Scopus, Web of Science, IEEE Xplore, and ScienceDirect.

All retrieved studies were imported into reference management software, and duplicate records were removed before screening. Two independent reviewers conducted title and abstract screening according to predefined eligibility criteria. Studies deemed potentially relevant were subsequently subjected to full-text review. Any disagreements between reviewers regarding study inclusion were resolved through discussion and consensus, and when necessary, a third reviewer was consulted (see Figure 1). Inter-reviewer agreement during the screening process was assessed using Cohen’s kappa coefficient, with a value of 0.80 indicating strong agreement.

Data extraction was performed independently by two reviewers using a standardized data extraction form. Extracted data included study characteristics, publication year, geographic location, study design, patient population characteristics, data type used for model development, AI methodology applied, model inputs, validation methods, outcome measures, and reported performance metrics. Any discrepancies in data extraction were resolved through discussion.

### 2.4. Study Outcomes

The primary outcomes of this systematic review were to evaluate the predictive accuracy of AI models using performance metrics such as sensitivity, specificity, and accuracy; the area under the receiver operating characteristic curve (AUC-ROC); and positive predictive value and negative predictive value. We also evaluated prognostic prediction using measures such as survival analysis (e.g., Kaplan–Meier curves, hazard ratios) or prognostic risk scores. In addition, recurrence prediction, including recurrence-free survival (RFS), time-to-recurrence metrics, and recurrence prediction accuracy, as well as treatment outcome prediction, were assessed.

### 2.5. Quality Assessment 

The methodological quality of the studies included in this review was assessed using the QUADAS-2 tool, a validated framework for evaluating diagnostic accuracy studies. This tool examines four key domains: patient selection, the index test, the reference standard, and flow and timing. Each domain is rated for risk of bias (high, low, or unclear), and the first three domains are also assessed for applicability concerns, which are also rated as high, low, or unclear. To guide these evaluations, signaling questions are used, such as, “Was a consecutive or random sample of patients utilized?” [17]. Additionally, we used the QUIPS (Quality in Prognostic Studies) tool, a critical appraisal instrument designed to assess the risk of bias in systematic reviews of prognostic factor studies. Developed through a rigorous methodology, it addresses the unique methodological challenges of prognostic research by assessing bias across six key domains: study participation, study attrition, prognostic factor measurement, outcome measurement, study confounding, and statistical analysis and reporting [18].

## 3. Results

### 3.1. Baseline Characteristics

Given the substantial heterogeneity in study design, data sources, AI algorithms, and reported outcomes, a quantitative meta-analysis was not feasible. Therefore, results were synthesized using the Synthesis Without Meta-Analysis Framework (SWiM), which grouped studies by their primary clinical application (prognosis prediction, recurrence prediction, and treatment outcome prediction). Key study characteristics and methodological details were summarized descriptively in structured tables, while performance metrics such as accuracy, AUC, sensitivity, specificity, and C-index were qualitatively compared across studies to identify patterns in model performance and clinical utility. Table 1 presents a comprehensive summary of the key features of the 29 studies featured in this systematic review. It highlights a pronounced inclination toward retrospective cohort designs and studies focused on diagnostic accuracy, using extensive databases such as Surveillance, Epidemiology, and End Results (SEER), National Cancer Database (NCDB), and various institutional datasets, with patient sample sizes ranging from 10 to more than 63,000. A consistent demographic observation across nearly all studies, including those by Choi et al. and Jones et al. was a significant male predominance, consistent with established epidemiological trends in LC [19,20]. Most research focused on LSCC, with a study by Wang et al. comparing glottic and non-glottic variants [21]. Various AI methods were employed, including traditional ML models like Random Forest and Extreme Gradient Boosting (XGBoost), as demonstrated by Alabi et al. for predicting survival rates [22]. Additionally, advanced DL techniques were used, such as the Swin-Transformer for image classification by Kang et al., and a specialized Super Resolution Enhanced-You Only Look Once (SRE-YOLO) model introduced by Baldini et al. for real-time lesion detection in endoscopic videos [4,23]. The input data utilized in these investigations was diverse, comprising Electronic Health Records (EHRs), medical imaging techniques such as CT scans and laryngoscopies, radiomic characteristics, and transcriptomic data sourced from the GEO database, as indicated by Du and Zu [2]. To enhance the reliability of their findings, researchers employed rigorous validation techniques, which included methods like train-test splits, k-fold cross-validation, and external validation using independent datasets. Wang et al. and Xu et al. demonstrated their models’ effectiveness by utilizing data from various centers to ensure that the findings are applicable across different populations [24,25].

### 3.2. Risk of Bias

**Figure 2:**
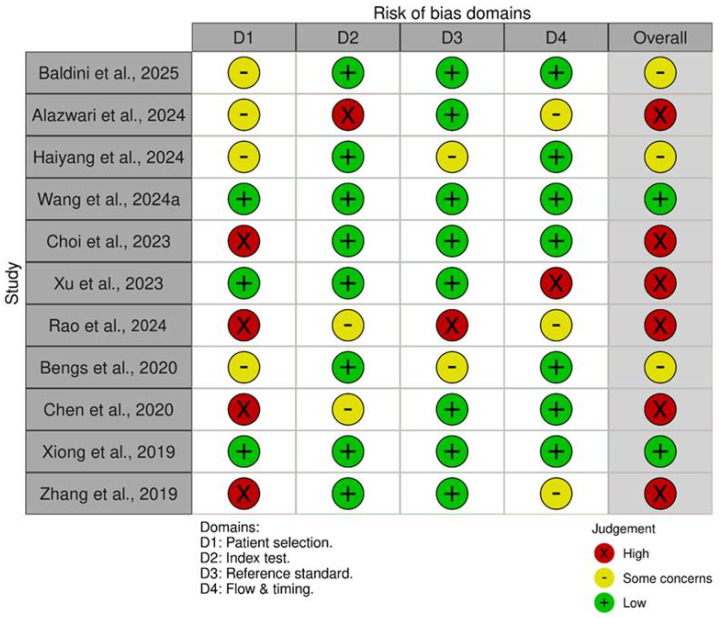
Risk of Bias Assessment Using QUADAS-2 [4,19,24,25,27,28,30,40,41,43,44].

Figure 2 displays the risk of bias across 11 diagnostic accuracy studies, evaluated using the QUADAS-2 tool. The assessment identified significant methodological issues, with most studies (8 out of 11) showing a high or unclear overall risk of bias. Only two studies (Baldini et al. and Bengs et al., demonstrated low risk across all domains [4,40]. The most common problems were in Domain 1 (Patient Selection) and Domain 2 (Index Test), indicating potential issues with study population recruitment and the execution or interpretation of the diagnostic tests being evaluated.

**Figure 3:**
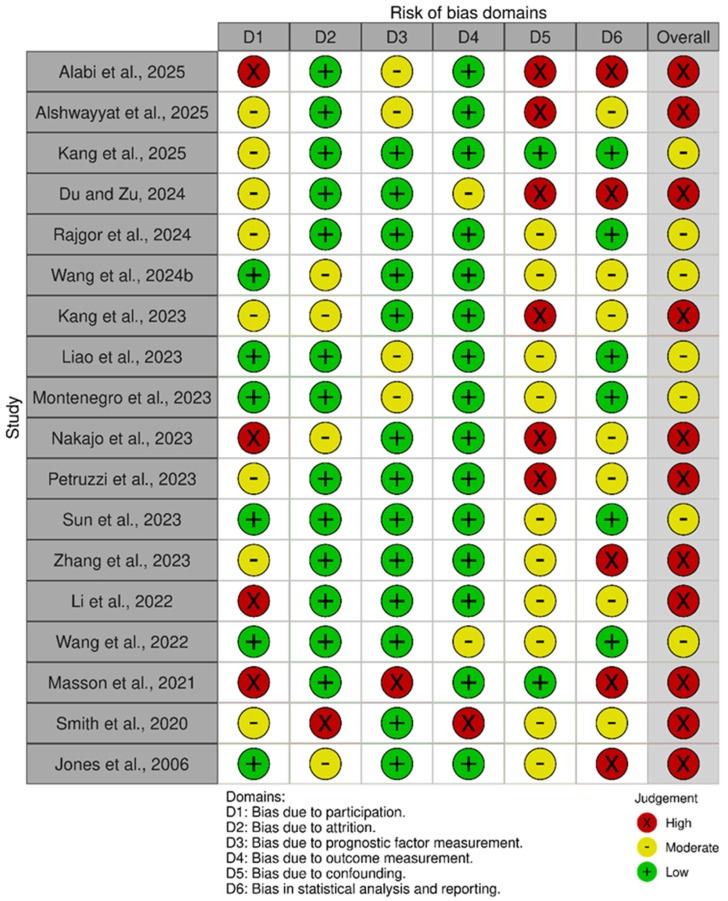
Risk of Bias Assessment Using QUIPS [2,3,20,21,22,23,26,29,31,32,33,34,35,36,37,38,39,42].

Figure 3 offers a detailed overview of bias risk across six methodological domains for 18 studies, highlighting variability in study quality. Most studies showed a low risk of bias in outcome and prognostic factor measurement areas. However, bias related to participation, attrition, and confounding was more prevalent. Notably, studies by Du and Zu, Kang et al., and Montenegro et al. consistently demonstrated low risk in all domains, indicating strong methodological rigor [2,23,33]. In contrast, studies by Alabi et al. and Zhang et al. yielded multiple high-risk judgments, suggesting potential limitations in internal validity [21,36].

### 3.3. Study Outcomes

Table 2 presents compelling evidence for the clinical utility of AI in the treatment of LC. The Predictive Accuracy Metrics reported across studies indicate that many AI models demonstrate diagnostically and prognostically useful performance in the context of LC. For instance, Li et al. reported an accuracy of 96.1% using a CNN applied to Raman spectroscopy data, highlighting the potential of AI-assisted spectroscopic analysis for accurate cancer detection [37]. Similarly, DL models developed for laryngoscopy image analysis, as demonstrated by Xu et al. and Kang et al., consistently achieved area under the curve (AUC) values exceeding 0.95 [23,25]. In addition, Baldini et al. demonstrated that the SRE-YOLO model achieved an average precision (AP) of 0.82 at an intersection-over-union (IoU) threshold of 0.5, indicating that it accurately detects and localizes LC lesions in real-time endoscopic images, thereby supporting clinicians during endoscopic evaluation [4].

Other imaging modalities have also shown promising results. Bengs et al.investigated the use of the DL architecture DenseNet3D combined with hyperspectral imaging for the detection of LSCC in vivo, achieving an average accuracy of 81% [40]. These findings indicate that integrating both spatial and spectral information can significantly enhance classification performance compared with conventional imaging approaches. Advances in molecular and histological analysis have also contributed to improved diagnostic capabilities. Du and Zu et al. reported that copper death-related genes (e.g., SLC31A1, ATP7B, ANXA5, SERPINH1) play an important role in LC prognosis, and demonstrated that ML algorithms such as Random Forest (RF) and Support Vector Machine (SVM) can effectively utilize these genetic markers to construct a diagnostic model, potentially facilitating earlier detection and more timely treatment [2]. Furthermore, multicolor stimulated Raman Scattering (SRS) microscopy enables label-free histological analysis of laryngeal tissues while providing diagnostic features comparable to traditional hematoxylin and eosin (H&E) staining [44]. Zhang et al. demonstrated that combining multicolor SRS imaging with the ResNet34 DL model enables rapid and accurate intraoperative diagnosis of fresh laryngeal tissue samples [44].

In the critical area of Prognosis Prediction, AI models often outperform traditional methods. Recent findings by Sun et al. indicated that the Random Survival Forest (RSF) model (C-index: 0.82–0.84) significantly outperformed the Cox regression model (C-index: 0.72–0.74) in predicting three-year survival rates [35]. Similarly, Choi et al. demonstrated that their Deep Neural Network (DNN) model achieved a C-index of 0.859, providing superior survival prediction compared to conventional statistical approaches, such as Cox proportional hazards (Cox-PH), RSF, and models relying solely on tumor-node (T/N) staging in patients with LSCC [19]. Notably, models based exclusively on T/N staging showed the lowest predictive accuracy, while incorporating multiple clinical variables through AI-based methods improved survival prediction and may support more informed clinical decision-making.

Further evidence supporting the prognostic value of AI-based approaches was reported by Zhang et al., who found that ML models, such as the RSF model and the Cox regression-based nomogram, demonstrated strong predictive performance for disease-free survival in advanced LSCC [36]. Key prognostic variables identified in this study included N stage, clinical stage, and postoperative chemoradiotherapy. Earlier work by Jones et al. also demonstrated the advantages of artificial neural networks (ANNs) over traditional statistical models [20]. In this study, the ANN model outperformed the Cox model in detecting survival differences, particularly for age and N stage, and produced a clear separation between risk groups. Although Kaplan–Meier analysis did not reveal statistically significant differences in overall survival, the neural network showed progressively increasing divergence in survival trends over time (χ^2^ for trend *p* = 0.0025), suggesting that ANNs are particularly effective in modeling complex and nonlinear relationships in survival data [20]. Additionally, according to Petruzzi et al., the decision-tree ML algorithm (J48) is highly effective in predicting 1- and 3-year oncological outcomes for surgically treated LC patients [3]. The study identified several prognostic factors: at 1 year, the Lymph Node Ratio (LNR) was the most influential predictor, whereas at 3 years, the number of metastases, perineural invasion, and grading were most significant [3]. In addition to survival prediction, some studies have focused on predicting metastatic spread, which may further support treatment planning. According to Wang et al., the decision-based fusion model (DLRad_DB), which integrates both radiomic features and clinical data, proved to be a highly effective tool for preoperative prediction of occult lymph node metastasis (LNM) in LC [30].

In the realm of recurrence prediction, several studies have proven the potential of AI-based methods to identify patients at risk of disease relapse. For example, Montenegro et al. and Zhang et al. successfully predicted recurrence by linking relapse risk to specific histopathological characteristics, including lymphovascular invasion (LVI), as well as clinical staging parameters [33,36]. Another remarkable advantage of these AI-driven models is their ability to predict treatment outcomes, thereby supporting clinical decision-making. For instance, the DeepSurv model could serve as an auxiliary tool to provide individualized treatment recommendations, such as identifying which patients are likely to benefit more from surgical intervention, according to Liao et al. [32]. Similarly, Masson et al. developed a radiomic model to predict non-responsiveness to induction chemotherapy, while Smith et al. applied a Gradient Boosting model to identify patients at risk of salvage laryngectomy following ineffective non-surgical treatment [39,42]. Moreover, the IRL tool developed by Wang et al. is designed to predict prognosis, assess immune infiltration levels, and evaluate responses to immunotherapy in patients with LSCC, thereby aiding tailored immunotherapy counseling [38].

Multiple studies have conducted comparative evaluations of different AI models. For example, comparative analyses consistently showed that RSF outperforms Cox proportional hazards (CoxPH), Gradient Boosting Machines (GBM), XGBoost, and DeepSurv, as demonstrated by studies such as Wang et al. [21]. Furthermore, Alabi et al. highlighted that ensemble ML techniques may outperform DL methods, like DeepTables, in handling structured tabular datasets, emphasizing the importance of selecting appropriate algorithms depending on the nature of the data [22].

## 4. Discussion

Laryngeal cancer (LC) is considered one of the most common types of HNC [2,4]. The diagnosis of LC is based on history, physical examination, imaging modalities, and histopathological confirmation [7]. However, despite improvements in LC diagnosis, conventional tools such as tumor-node-metastasis (TNM) staging often exhibit limited predictive accuracy for individual outcomes [8]. Recently, AI models have emerged as promising tools in oncology, demonstrating improved capabilities in survival prediction, diagnostic accuracy, early detection, and treatment response assessment [10,11,12]. Therefore, the objective of this systematic review was to investigate the role of AI in determining the prognosis, predicting recurrence, and assessing treatment outcomes in laryngeal cancer.

In this review, AI demonstrated strong prognostic accuracy for predicting treatment outcomes and recurrence in laryngeal cancer. Across the included studies, ensemble machine-learning models such as the Random Survival Forest (RSF) consistently outperformed traditional prognostic methods, including Cox regression and TNM staging. These findings align with those reported by Davenport T. et al. and Hosny A. et al., who noted that, compared to older computer-aided detection tools, combining deep learning (DL) with radiomics in analyzing cancerous lesions can significantly improve diagnostic accuracy and support clinical decision-making in cancer risk stratification [45,46,47]. Additionally, our findings show that AI models can identify important recurrence- and prognostic-related features, such as lymphovascular invasion (LVI) and cartilage infiltration, as significant prognostic factors. In addition to prognostication, AI demonstrated utility in treatment decision-support, including prediction of chemotherapy response and the future risk of salvage laryngectomy. Furthermore, Smith et al. found that machine learning (ML) models could predict which patients might need surgical intervention, though their predictive performance depended on the quality of clinicopathological data from the NCDB [42]. These findings highlight the importance of improving the availability and quality of clinical data to maximize the predictive accuracy of AI-based models in laryngeal cancer.

Furthermore, DL models trained on large multi-institutional cohorts have successfully identified genomic correlates of imaging-derived features, linking radiologic phenotypes to underlying biological processes such as cell-cycle regulation and transcriptional activity [48]. These parallels suggest that integrating radiomic, genomic, and clinical data could similarly enhance predictive accuracy in laryngeal cancer, particularly given that multi-omic approaches remain underutilized in this disease.

Several included papers demonstrated that ensemble approaches generally yield superior predictive performance compared with single-algorithm models. For example, Alabi et al. found that XGBoost, Voting, and Stacking models more accurately predicted overall survival than DeepTables, whereas Alshwayyat et al. showed that Random Forest and Gradient Boosting models outperformed K-nearest Neighbors [22,23]. Similarly, Nakajo et al. reported that the Naïve Bayes model was effective at predicting recurrence, whereas RSF performed better at predicting progression-free survival (PFS). In another study, Petruzzi et al. suggested that both the Naïve Bayes classifier and the J48 decision tree were similarly effective at predicting outcomes [3,34]. These observations are consistent with findings in lung cancer, where AI-driven radiomic models incorporating both intratumoral and peritumoral imaging features have demonstrated strong prognostic performance, with reported C-index exceeding 0.72 and outperforming conventional TNM staging alone [48]. Collectively, these findings highlight the importance of algorithm selection and suggest that ensemble-based approaches may yield improved predictive performance on heterogeneous clinical datasets.

AI applications in LC have expanded to include multimodal data integration, including radiomic, genomic, and imaging datasets. Evidence from HNC research indicates that combining genomic and radiomic data improves prognostic accuracy compared with clinicopathological variables alone [49]. According to Rajgor et al. (2024), AI models built with radiomic data–either alone or combined with clinical variables–performed better than those based solely on clinicopathological factors in advanced laryngeal cancer [29]. Consistent with this, most studies in this review relied on radiomic and clinical data, while genomic integration remains limited. Emerging evidence suggests that genomic data may further enhance predictive models. For example, Du and Zhu (2024) demonstrated that copper death-related genes can be used in ML techniques to develop sensitive diagnostic tools for LC [2]. Additionally, in lung cancer, DL models trained on large multi-institutional cohorts have successfully identified genomic correlates of imaging-derived features, linking radiologic phenotypes to underlying biological processes such as cell-cycle regulation and transcriptional activity [48]. Comparable advancements in healthcare data analytics, including applications during the COVID-19 pandemic, have demonstrated the effectiveness of integrating imaging data with advanced neural network architectures [50]. Furthermore, high-dimensional inference methods, including the Split and Smoothing for High-Dimensional Inference framework, have demonstrated the ability to identify significant predictors from complex genomic datasets [50]. Together, these findings highlight the potential of multi-omic integration to improve predictive performance in laryngeal cancer, an area that remains underexplored.

This systematic review is supported by a large sample size and diverse data sources, including SEER, NCDB, TCGA, GEO, multicenter imaging cohorts, and multiple laryngoscopy image datasets. These heterogeneous datasets enable a comprehensive evaluation of AI performance across diverse patient populations and clinical settings, thereby increasing the generalizability of the results. Furthermore, the studies included in this review used rigorous validation techniques, such as k-fold cross-validation, which enhance the reproducibility and reliability of the reported findings. However, most validation techniques were internal, with relatively few studies performing external validation using independent datasets. This limitation is consistent with findings from a systematic review on HNCs, which reported that AI applications in this field are often constrained by variability in data collection methods and a lack of external validation, potentially limiting the generalizability of the developed models [48]. A further critical limitation, common across AI research in oncology and beyond, is the underrepresentation of diverse populations in training datasets. When data are limited or not representative of demographic diversity, AI models risk producing unfair predictions or systematically biased treatment recommendations [51,52,53]. Srivastav et al. (2025) demonstrated that AI models trained mainly on non-diverse datasets show reduced generalizability and may continue existing healthcare disparities, especially for underrepresented racial and ethnic groups [53]. An upcoming and promising solution is federated learning, where models are trained across multiple institutions without sharing raw patient data—offering a promising way to improve both data diversity and privacy [49,50,53]. The implementation of federated learning frameworks in laryngeal cancer research could directly address the external validation gap in this review, enhance the demographic representativeness of trained models, and position laryngeal cancer AI research within the broader trajectory of equitable, multi-institutional precision oncology.

Several limitations should be recognized. First, most of the included studies used retrospective cohort designs, which are naturally prone to selection bias, incomplete data collection, and differences in data quality, potentially affecting model performance and limiting causal conclusions. Second, there was significant variability across studies regarding data sources, study populations, AI algorithms, and outcome measures. Studies used multiple data sources, including imaging data, EHRs, radiomic features, and genomic information, while applying various ML and DL techniques such as CNNs, RSF, gradient boosting models, and DNNs. This methodological variety hindered the direct comparison of findings and limited the possibility of conducting a quantitative meta-analysis. Standardization represents an equally pervasive cross-domain challenge. In radiology AI, variability in imaging acquisition protocols, feature extraction pipelines, and outcome definitions has necessitated the development of structured evaluation frameworks such as radiomic quality scoring systems, as well as international initiatives including the Image Biomarker Standardization Initiatives [48]. Similarly, in large-scale healthcare data analytics, the absence of harmonized data platforms and consistent methodological reporting has been identified as a significant barrier to reproducibility and cross-institutional knowledge transfer [50]. These concerns are directly applicable to the laryngeal cancer AI literature, where the lack of standardized data collection protocols and outcome reporting represents one of the most important obstacles to the comparability and clinical translation of study findings. Moreover, challenges related to model interpretability and standardization further compound these limitations and have been consistently identified across multiple domains, including oncology and infectious disease research. Black-box DL architectures remain a key limitation, as their opacity impedes clinicians’ trust, regulatory approval, and clinical adoption. In response, explainable AI techniques-including Shapley Additive Explanations, gradient-weighted class activation mapping, and saliency maps-have been developed to improve transparency in model decision-making by attributing predictions to specific imaging features or clinical variables [49,53]. The adoption of these approaches in laryngeal cancer would enhance clinician trust and facilitate regulatory approval, particularly as AI systems are intended to function as decision-support tools rather than a replacement for clinical judgment.

Additional methodological limitations were also noted. Many studies in the diagnostic accuracy field showed high or unclear risk of bias, especially in patient selection, indicating that the study population might not fully represent a real-world clinical setting. Likewise, several prognostic studies displayed moderate to high bias risk due to confounding factors, which could impact the reliability of outcome predictions. Furthermore, the performance of AI methods depends heavily on the quality and completeness of input data, underscoring the need for standardized, high-quality clinicopathological datasets.

Beyond methodological considerations, several ethical issues must be considered when integrating AI into clinical practice, as discussed by Bergquis et al. [51] and Obuchowicz et al. [52]. One of these considerations is the need for strong regularity frameworks to ensure the safe implementation of AI systems, as premature deployment without adequate oversight may lead to unreliable recommendations and potential patient harm. Responsibility and reliability also remain critical concerns, since AI systems are human-developed tools and clinicians must retain final accountability for clinical decisions and patient outcomes [49,50]. Ethical use of large-scale medical data is another important consideration, necessitating secure data management, responsible data sharing, and strict protection of patient confidentiality [49]. Furthermore, AI systems may also produce erroneous or hallucinated outputs, reinforcing the need for continuous physician oversight and verification [50]. Collectively, these issues highlight that AI should function primarily as a decision-support tool that augments, rather than replaces, human clinical judgment, ensuring that patient-centered care and ethical medical practice remain central in the management of laryngeal cancer [49,50].

Taken together, these limitations emphasize the need for prospective studies, standardized data collection methods, and consistent reporting of AI model performance. Such efforts will be essential to improve comparability between studies and facilitate the integration of AI tools into routine clinical practice for prognosis prediction, recurrence risk assessment, and treatment outcome evaluation in patients with laryngeal cancer.

## 5. Conclusions

In conclusion, the findings presented in this review suggest that AI has the potential to support the diagnosis and management of LC. AI-based approaches may improve the analysis of complex clinical and imaging data and assist clinicians in identifying patterns related to diagnosis, prognosis, and treatment planning. However, while AI may serve as a useful adjunct in clinical decision-making, further prospective research, external validation, and standardized evaluation are required before these technologies can be reliably integrated into routine clinical practice.

## Figures and Tables

**Table 1:** Baseline Study and Patient Characteristics of Included Studies.

First Author (Year)	Study Design	Sample Size	Age (Mean/Median ± SD or Range, Years)	Gender	Laryngeal Cancer Subtype and Stage	Histopathological Confirmation	AI Model Details	Data Input Used	AI Model Validation Performed
Alabi et al., [22]	Retrospective Cohort Study	2792 patients	Mean: 65.3 (11.1) years Range: 21–90 years	M: 2261 F: 531	Subtype: LSCC Stage (AJCC 7th ed.): • T-stage: T1 (31.8%), T2 (27.8%), T3 (24.1%), T4 (16.3%) • N-stage: N0 (69.5%), N1 (10.3%), N2 (19.1%), N3 (1.1%) • M-stage: M0 (97.4%), M1 (2.6%)	Yes, histologically confirmed SCC of the larynx	1. Ensemble Methods: Voting Ensemble, Stack Ensemble, XGBoost 2. DL: DeepTables Best Performing Models: Ensemble methods (Voting, Stack, XGBoost) outperformed DeepTables.	EHRs	DeepTables used a 50:50 train-test split. Ensemble models used 5-fold cross-validation.
Alshwayyat et al., [23]	Retrospective Cohort Study	63,324 patients	Median age: 62 years. Groups: <50 yrs: 10.2% (6488), 50–60 yrs: 26.6% (16,844), >60 yrs: 63.2% (39,992)	M: 51,197 F: 12,127	Subtypes: - Glottic: 40,824 (64.5%) - Supraglottic: 21,774 (34.4%) - Subglottic: 726 (1.1%) Stage: - Localized: 72.5% (45,904) - Regional: 27.5% (17,420)	Yes	Five ML Models: 1. LR 2. KNN 3. RFC 4. GBC 5. MLP Task: Binary classification to predict “5-year survival”	EHRs	Yes - Train/Test split (80/20) - Tenfold cross-validation - Mean Bootstrap Estimate with 95% CI - ROC/AUC analysis
Baldini et al., [4]	Retrospective multicenter study	- Images: 5852 (3721 internal + 149 external) - Patients: 1593 (internal datasets)	NR	NR	Subtypes: Malignant (LSCC) and Benign (cysts, granuloma, leukoplakia, papilloma, polyps, Reinke’s edema, nodules).	NR	Name: SRE-YOLO Architecture: YOLOv8n (nano) baseline with a decoupled Super-Resolution (SR) branch attached during training. The SR branch uses an EDSR-like decoder.	WL and NBI endoscopic images/frames.	- Internal validation with patient-level split (Training/Validation/Test). - External validation on the separate “ENDO-LC ext” dataset. - Multiple hold-out splits for robustness testing.
Kang et al., [26]	Diagnostic accuracy study	1462 patients 5768 images	NR	NR	Normal: 718 Benign lesions: 375 Precancerous Lesions: 106 Cancer: 263	Yes	Name: ILCDS Type: Swin-Transformer (a self-attention-based DL model).	Laryngoscopy images.	Yes, Internal validation (train/validation/test split) and external validation on a dataset from a different medical center.
Alazwari et al., [27]	Retrospective Observational Study	1320 image patches from 33 unique patients	NR	NR	Laryngeal spinocellular carcinoma; early-stage cancerous tissues	Yes	LCD-CMDL Technique: 1. Preprocessing: CLAHE 2. Feature Extraction: SE-ResNet 3. Hyperparameter Tuning: CSSA 4. Classifier: ELM	Medical Imaging (CT scans, Laryngoscopic Images)	Yes, hold-out validation (80% training/20% test and 70% training/30% test)
Du and Zu, [2]	Bioinformatic Analysis Study	Total: 109 samples from two GEO datasets. Cancer Tissues: 87 (56 from GSE25727 + 31 from GSE27020 training/validation sets, based on typical dataset splits). Normal Tissues: 22 (from GSE25727 control group).	NR	NR	LSCC	Yes, all samples were from “human laryngeal cancer specimens” and “microscopic confirmation.	AI Models: Four ML algorithms were used: GLM, XGB, SVM, and RF.	Gene expression data (transcriptomes) from the GEO database.	Validation Method: LOOCV and Bootstrap internal validation.
Wang et al., [28]	Retrospective Study on Radiomics Feature Augmentation	11,144 images from 210 adult patients	NR	NR	NR	Yes	Type of AI: Generative Adversarial Network (CTGAN) and ML Models (SVM, RF, XGBoost) Specific AI: CTGAN for radiomics feature augmentation; SVM, RF, XGBoost for classification Data input: Medical Imaging (NBI)	Medical Imaging, NBI	Validation of Synthetic Data Quality Tool Used: SDV library (version 0.18.0) in Python (Python 3.10)
Rajgor et al., [29]	Retrospective cohort study	72	Median 66 years (range 59–71)	M: 53 F: 19	Advanced LSCC (T3–T4)	Yes, all confirmed via biopsy	Radiomic feature extraction via LIFEx; LASSO-based Cox regression	Pre-biopsy contrast-enhanced CT scans	Yes, internal validation via cross-validation
Rao et al., [24]	Retrospective Diagnostic Study	88 patients (from the initial 277 after exclusions)	NR	NR	TNM stages included, Majority T1–T5	Yes	Three models compared: 1. Logistic Regression: Baseline interpretable model 2. 4-Layer Neural Network: Moderate complexity with ReLU/sigmoid activation 3. ResNet-50: 50-layer CNN with transfer learning, pre-trained on a large dataset	Medical imaging	Yes, Training/validation/test split with performance evaluation on the validation set
Wang et al., [30]	Multicentre, Retrospective, Diagnostic Study	553 patients (300 training, 89 internal test, 120 external test set 1, 44 external test set 2)	61 (8) years	M: 532 F: 21	Subtype: LSCC—Glottic (27%) and Supraglottic (73%). Stage: Clinical N0 stage (no lymph node metastasis detected clinically). T stages: T1 to T4.	Yes, Pathological verification of LSCC.	- Radiomics Model: 1834 features extracted by PyRadiomics, feature selection (LASSO), SVM classifier. - 2D DL: ResNet50 (pretrained on ImageNet) fine-tuned, using the maximum tumor cross-section and 6 adjacent slices. - 3D DL: 3D ResNet50 (pretrained on Med3D) fine-tuned, using the 3D tumor volume. - Fusion Models: - DLRad_FB (Feature-based): Combination of radiomics, 2D DL, 3D DL, and clinical features. - DLRad_DB (Decision-based): Stacking of the four base models (radiomics, 2D DL, 3D DL, clinical) using a random forest.	Medical Imaging (CT Scans), Radiomics, Clinical Data (EHRs)	Yes. Internal validation (89 patients) and external validation on two independent sets (120 and 44 patients).
Wang et al., [21]	Retrospective Observational Study	10,418 Glottic: 5953 patients; Non-Glottic: 4465 patients	Training cohort: 63 years (Range: 56–70). Validation cohort: 63 years (Range: 57–70)	Training cohort: Male: 86.9%, Female: 13.1%. Validation cohort: Male: 88.8%, Female: 11.2%	Subtypes: Glottic vs. Non-Glottic (Supraglottic and Subglottic). Stages: I, II, III, IVa, IVb, IVc (AJCC 7th Edition)	Yes, data from the SEER cancer registry	Models Tested: CoxPH, RSF, GBM, XGBoost, Deepsurv.	Clinico-pathological variables from the SEER database (age, sex, histology, tumor size, AJCC T/N/M stage, treatment-surgery, radiotherapy, chemotherapy)	Yes. 10-fold cross-validation and a hold-out test set (10% of data).
Choi et al., [19]	Retrospective Observational Single-Center Study	1020 patients	64.5 ± 9.7 years	M: 979 F: 41	Subtypes: Supraglottic (22.6%), Glottic (75.2%), Subglottic (0.22%). T Stage: T1 (58.0%), T2 (19.5%), T3 (13.3%), T4 (9.1%) Overall Stage: I (56.7%), II (14.1%), III (9.5%), IV (19.7%)	Yes	Models: DNN-MC, DNN-Reg, RSF, CoxPH	Clinical variables (age, sex, smoking, alcohol, tumor location, TNM stage, treatment, recurrence)	Yes, 5-fold cross-validation.
Kang et al., [31]	Retrospective Cohort Study	114 patients (Training: *n*= 81, Validation: *n* = 33)	Training: PR group 46.7 ± 10.2, Non-PR 47.8 ± 11.3 Validation: PR group 43.7 ± 9.2, Non-PR 42.8 ± 7.3	M: 95 F: 19	Subtypes: Supraglottic and Glottic. Stage: Locally Advanced (T3–T4a, N0–N3)	Yes	Model Type: Radiomics Signature (rad-score) built using LASSO regression. Purpose 1 (PR): LR. Purpose 2 (OS): CoxPH model integrated into a Nomogram.	Clinico-pathological features (Age, Gender, Tumor Site, T/N Stage, Tumor Size/Volume, Radiation) and 851 radiomic features from pretreatment contrast-enhanced CT images.	Yes. Internal validation via a randomly split training/validation cohort (7:3 ratio).
Liao et al., [32]	Retrospective Cohort Study	Total: 6316 (Development: 4237; Validation: 2079)	Median (IQR): Development: 63 (56–70) years Validation: 63 (57–70) years	Development: M: 3350 F: 887 Validation: M: 1632 F: 447	Subtypes: Glottic (37.0%), Supraglottic (48.1%), Subglottic (2.3%), Others (12.6%). Stages: I (17.8%), II (13.9%), III (22.4%), IV (46.0%)	Yes (Required for inclusion from SEER database)	Name: DeepSurv Type: DL, fully connected feed-forward neural network. Architecture: Input layer (23 nodes), 3 hidden layers (30, 20, 20 nodes, ReLU activation), 1 output node. Function: Cox proportional hazards deep neural network.	Structured clinical data from the SEER database (sex, age, stage, grade, tumor site, treatment details).	Yes. Internal validation via a held-out test set (80/20 split from the development cohort) and a separate temporal validation cohort. 5-fold cross-validation and bootstrapping (1000 iterations) were used for hyperparameter tuning and robust performance estimation.
Montenegro et al., [33]	Retrospective prognostic study	74 patients	Mean: 69 years Range: 38–93 years	M: 69 F: 5	pT4a LSCC pN Category: pN0: 63.5%, pN1: 6.8%, pN2b: 4.1%, pN3b: 24.3%	Yes, all cases postoperative histopathology	Model Types: Multiple ML algorithms Algorithms Used: LR, KNN, SVM (Linear and RBF), Gaussian NB, Decision Tree Classifier, RFC	Medical Imaging (CT, MRI), Radiomics, EHRs	Yes, Method: Training-validation split
Nakajo et al., [34]	Retrospective Cohort Study	49 patients (Training: *n* = 34, Testing: *n* = 15)	Mean: 72 ± 11 years; Range: 44–96 years	M: 46 F: 3	Subtype: SCC (all patients). Stage: I (*n* = 13), II (*n* = 7), III (*n* = 13), IVA (*n* = 15), IVB (*n* = 1)	Yes	For Disease Progression (Binary Classification): RF, Neural Network, KNN, NB, LR, SVM	Clinical features (age, sex, tumor size, T/N/UICC stage, treatment) and 40 radiomic features from pretreatment 18F-FDG-PET/CT images.	Yes. Split into training (70%) and testing (30%) cohorts. Used 10-fold cross-validation and feature reduction to mitigate overfitting.
Petruzzi et al., [3]	Retrospective Cohort Study	132 patients	Mean: 62 years (Range: 19–88)	M: 107 F: 25	Subtypes: Glottic (71.2%), Supraglottic (28.0%), Subglottic (0.7%). Stages (Post-op): I (4.5%), II (18.2%), III (31.1%), IVA (32.6%), IVB (13.7%)	Yes, Squamous cell carcinoma was confirmed in all patients	J48 algorithm (a decision tree classifier, implementation of C4.5) in the Weka data mining tool.	Clinical, surgical, and pathological variables from patient records (age, subsite, type of surgery, pT/pN stage, grading, LNR, adverse features).	Yes, Internal validation via a 70/30 split (70% for training, 30% for testing). Multiple random seeds were tested to ensure robustness of the results.
Sun et al., [35]	Observational cohort study	8677 patients	NR	NR	Subtype: LSCC Stage: AJCC overall stage, T stage, and N stage	Yes (ICD-O-3 codes 8050–8089 for squamous cell neoplasms; microscopically confirmed)	AI Model: RSF Software/Package: R package random Forest SRC, version 3.2.0 (https://cran.r-project.org/package=randomForestSRC, accessed 10 February 2023) Parameters: ntree = 1000, mtry = 12, nodesize = 15, nsplit = 10	Demographic, clinical, and treatment data from the SEER database.	Yes, Validation Method: 70:30 split into training and validation sets. Metrics: C-index, AUC, Brier score, calibration plots.
Xu et al., [25]	Diagnostic accuracy multi-centre study	428 patients (2254 laryngoscopic images)	Medical Center A (Training/Internal Validation): Benign: 45 ± 12.3 years (training), 46 ± 12.8 years (internal validation) Malignant: 52 ± 8.6 years (training), 52 ± 9.6 years (internal validation) Medical Center B (External Validation): Benign: 41 ± 11.2 years Malignant: 53 ± 9.1 years	Training Cohort: Benign: 53M/74F; Malignant: 102M/3F Internal Validation: Benign: 20M/33F; Malignant: 44M/1F External Validation: Benign: 24M/29F; Malignant: 44M/1F	LSCC	Yes, all cases are biopsy-proven	Primary Model: Densenet201 Architecture: Densely Connected Convolutional Networks Technical Specifications: Hardware: NVIDIA RTX 3090 24G Batch Size: 64 Learning Rate: 0.001 (decreased by 0.5 every 100 epochs) Total Epochs: 16,000 Image Resolution: 512 × 512 Optimizer: SGD	WL laryngoscopic images	AI Model Validation Performed: Yes Methods: Internal validation + external validation (multi-center) Comparison: Two clinicians (30 years and 10 years of experience)
Zhang et al., [36]	Retrospective Cohort Study	671 patients	≤60 years (*n* = 336) and >60 years (*n* = 335).	M: 655 F: 16	Subtypes: Supraglottic (36.5%), Glottic (62.1%), Subglottic (1.3%). Stages: III (64.7%), IVa (33.1%), IVb (2.2%)	Yes	Name/Type: RSF Specifications: 500 trees.	Clinico-pathological data from a single institution (T/N stage, clinical stage, tumor volume, resection margins, treatment details).	Yes. Internal validation via a 70/30 split (70% for training, 30% for validation).
Li et al., [37]	Diagnostic Accuracy Study	10 human laryngeal cancer surgical specimens	NR	NR	NR	Yes	Models: RF and 1D-CNN Task: Binary classification of Raman spectra into “normal” or “tumor.”	Spontaneous Raman Spectroscopy signals (440 wavenumbers from 509 to 3978 cm^−1^)	Yes, 90% for training, 10% for testing. Results averaged over 10 trials (RF) and 50 trials (CNN).
Wang et al., [38]	Retrospective Cohort Study	TCGA: 109; GEO: 48–109	NR	NR	All stages of LSCC	Yes (TCGA/GEO)	IRL classifier using LASSO + RF; 3 lncRNAs	RNA-seq, clinical data, mutation data	Yes, external validation via GEO datasets
Masson et al., [39]	Multicenter Retrospective Observational Study	98 patients	Median (IQR): Training set: 59.5 (9), Testing set: 60.5 (10)	Training: M: 57 F: 9 Testing: M: 27 F: 5	Locations: Supraglottic, Glottic, Hypopharynx. T Stages: T2, T3 (with/without cord fixation), T4a	Yes, histologically proven	Method: Radiomics. Feature Types: Intensity, Shape, Textural (GLCM, NGTDM, GLRLM, GLSZM). Harmonization: ComBat with unsupervised clustering.	Medical Imaging (Contrast-Enhanced CT scans—CE-CT)	Yes. Split into Training/Validation (*n* = 66) and Testing (*n* = 32) sets.
Bengs et al., [40]	Retrospective Diagnostic Accuracy Study	100 patients • 70 patients had both healthy and tumor areas marked. • 30 patients had only a tumor area marked.	NR	NR	NR	Yes	Architecture: DenseNet Three variants were tested: 1. Densenet2D: Uses 2D convolutions; 30 spectral channels as input channels. 2. Densenet2D-MS: Uses 2D convolutions; input is a 2-channel image (pixel-wise Mean and Standard Deviation of spectral dimension). 3. Densenet3D: Uses 3D spatio-spectral-convolutions; input is a 3D cube (32 × 32 × 30).	Medical Imaging	Yes. 8-fold cross-validation. Data was split into test and validation subsets for each fold. The validation subset was used for hyperparameter tuning.
Chen et al., [41]	Retrospective Cohort Study	136 patients (Training: *n* = 96, Validation: *n* = 40)	Median: 60 years; Range: 30–86 years	M: 128 F: 8	Subtype: SCC Location: Supraglottic (*n* = 30), Glottic (*n* = 102), Subglottic (*n* = 4). Stage: I (*n* = 38), II (*n* = 39), III (*n* = 38), IV (*n* = 21)	Yes, for all patients	Model Type: Radiomics Signature (Rad-score) built using LASSO-Cox regression. Final Model: A radiomics nomogram incorporating the Rad-score and significant clinical factors (tumor location, T/N stage, laryngectomy type) using a multivariate Cox model.	Clinico-pathological features (age, gender, tumor location, T/N stage, histological grade, laryngectomy type) and 36 radiomic features from preoperative contrast-enhanced CT images.	Yes. Internal validation via a randomly split training/validation cohort (7:3 ratio).
Smith et al., [42]	Retrospective Cohort Study	16,440 patients (Training: *n* = 13,153, Validation: *n* = 3287)	No STL: 62.4 ± 10.5 years Yes STL: 61.2 ± 9.9 years	M: 12,275 F: 4175	Subtype: SCC Subsite: Glottis, Supraglottic, Other/Unspecified. Stage: cT1-T3a, all N stages (N0–N3), M0.	Yes, implied by database coding for SCC, though specific pathological	Algorithms Tested: LR, Decision Forest, Kernel Support Vector Machines, Gradient Boosting. Best Model: Gradient Boosting.	Sociodemographic and healthcare quality factors from the NCDB. Clinical T/N stage, laryngeal subsite, Charlson Comorbidity Index, days from diagnosis to treatment, distance to treating facility, age, facility type, sex, insurance type, income, and education level.	Yes. Data was split into an 80/20 training-validation set. Used 5-fold cross-validation during training and recursive feature elimination. The training set was up-sampled to handle class imbalance.
Xiong et al., [43]	Diagnostic accuracy multicenter study	14,897 images from 2208 subjects • Training: 13,721 images from 1816 subjects • Testing: 1176 images from 392 subjects	NR	NR	Laryngeal cancer	Yes	GoogleNetInception v3 with transfer learning • Pre-trained on ImageNet • Batch size: 64, Learning rate: 0.001 • Data augmentation factor: 720 • Hardware: 4 Titan XP 12 GB GPU	White light laryngoscopic images	Yes, Internal and external validation DS1 + DS2 training DS3 testing (external validation)
Zhang et al., [44]	Diagnostic accuracy study	78 patient cases (45 for training/validation, 33 for testing)	NR	NR	LSCC	Yes	Model: 34-layered Residual CNN (ResNet34). Input: 200 × 200 pixel image tiles from SRS maps.	Medical Imaging (Stimulated Raman Scattering Microscopy)	5-Fold Cross-Validation on the training set (18,750 image tiles). • Hold-out Testing on an independent set of 33 fresh surgical specimens.
Jones et al., [20]	Retrospective cohort study	873 patients with LSCC were randomly divided into the training set (*n* = 436) and the study set (*n* = 437)	Mean age: 62.5 years (all patients) • Training group: 62.3 years • Study group: 62.7 years	Male: 680 Female: 193	Subsites: • Glottic: 406 (46.5%) • Supraglottic: 419 (48.0%) • Subglottic: 48 (5.5%) T Stage: • T1-2: 548 (62.8%) • T3-4: 325 (37.2%) N Stage: • N0: 702 (80.4%) • N1-3: 171 (19.6%)	Yes	Model Type: Artificial Neural Network (ANN) Software: MATLAB (version 6) Architecture: Single hidden layer with three nodes	EHRs	Yes—Internal validation using train-test split (50-50 random division) • Confirmed groups were well-matched (*p*-values 0.1884–0.6026) • Network run 10 times for each covariate to calculate 95% CIs

LSCC: Laryngeal Squamous Cell Carcinoma, XGBoost: Extreme Gradient Boosting, AJCC: American Joint Committee on Cancer, RFC: Random Forest Classifier, GBC: Gradient Boosting Classifier, EDSR: Enhanced Deep Residual Network for Single Image Super Resolution, WL: White Light, NBI: Narrow Band Imaging, ILCDS: Intelligent Laryngeal Cancer Detection System, SE-ResNet: Squeeze-and-Excitation ResNet, CLAHE: Contrast Limited Adaptive Histogram Equalization, CSSA: Chaotic Adaptive Sparrow Search Algorithm, ELM: Extreme Learning Machine, ANN: Artificial Neural Network, CE-CT: Contrast-Enhanced Computed Tomography, CNN: Convolutional Neural Network, CT: Computed Tomography, DNN: Deep Neural Network, EHRs: Electronic Health Records, GEO: Gene Expression Omnibus, GBM: Gradient Boosting Machine, GLM: Generalized Linear Model, GAN: Generative Adversarial Network, CTGAN: Conditional Tabular GAN, KNN: K-Nearest Neighbors, LCD-CMDL: Laryngeal Cancer Detection using Chaotic Metaheuristics with Deep Learning, DL: Deep Learning, LOOCV: Leave-One-Out Cross-Validation, LR: Logistic Regression, ML: Machine Learning, MLP: Multilayer Perceptron, NCDB: National Cancer Database, NR: Not Reported, RF: Random Forest, RSF: Random Survival Forest, RT: Radiotherapy, SCC: Squamous Cell Carcinoma, SD: Standard Deviation, SEER: Surveillance, Epidemiology, and End Results, SRE-YOLO: Super-Resolution Enhanced YOLO, SVM: Support Vector Machine, TCGA: The Cancer Genome Atlas, TNM: Tumor, Node, Metastasis, WGCNA: Weighted Gene Co-expression Network Analysis, XGB: XGBoost, LASSO: Least Absolute Shrinkage and Selection Operator, LIFEx: Local Image Feature Extraction, CoxPH: Cox Proportional Hazards, DNN-MC: Deep Neural Network with Multi-classification, DNN-Reg: DNN with Regression, NB: Naive Bayes, DenseNet: Densely Connected Convolutional Networks.

**Table 2:** AI Model Performance, Predictive Outcomes, and Comparative Effectiveness.

First Author (Year)	Predictive Accuracy Metric	Prognosis Prediction	Recurrence Prediction	Treatment Outcome Prediction	Comparative Effectiveness of AI Models	Conclusion
Alabi et al., [22]	Best Performers (Ensemble Models): • Accuracy: 71.8% (Voting & XGBoost), 70.2% (Stack) • Weighted AUC: 76.9 (Voting), 76.8 (Stack), 76.1 (XGBoost) • Sensitivity/Recall: 0.66 (Voting & XGBoost), 0.69 (Stack) • Specificity: 0.75 (Voting & XGBoost), 0.70 (Stack) • Precision: 0.60 (Voting & XGBoost), 0.45 (Stack)	Prediction probability for “Low survival (Dead)”: 0.52 (52%) Prediction probability for “High survival (Alive)”: 0.48 (48%	NR	NR	Tree-based ensemble methods (XGBoost, Voting, Stack) significantly outperformed the DL model (DeepTables) for this tabular data task.	The study concludes that ML ensemble models are effective and outperform DL for predicting overall survival in laryngeal cancer using clinical data. Key prognostic factors identified were age, N-stage, T-stage, tumor grade, and marital status.
Alshwayyat et al., [23]	- Accuracy: Ranged from 54.84% (KNN-Glottic) to 71.76% (RFC/GBC-Subglottic) - Precision: Ranged from 48.00% to 64.78% - Recall: Ranged from 5.88% (LR-Supraglottic) to 73.37% (MLP-Glottic) - F1-Score: Ranged from 10.65% to 64.14% - AUC: Ranged from 0.5800 (KNN-Glottic) to 0.7060 (RFC/MLP-Subglottic)	Predicted 5-year Overall Survival (OS) and Cancer-Specific Survival (CSS). Identified key prognostic factors (age, stage, tumor size) for each cancer subtype.	NR	Yes. Comprehensive analysis of how different treatments (Surgery, RT, CTX, combinations) affect 5-year OS and CSS rates for each cancer subtype.	Yes. Direct comparison of five different ML algorithms, showing that ensemble methods (RFC, GBC) generally outperformed others, especially for subglottic cancers.	The study underscores the critical role of individual factors (age, stage, tumor size) in the management of non-metastatic LSCC. Surgery, often combined with radiotherapy, was identified as the most effective approach. ML models, particularly RFC and GBC, effectively predicted 5-year survival. A web-based tool was developed to facilitate clinical decision-making.
Baldini et al., [4]	Primary Metric: Average Precision at IoU = 0.5 - SRE-YOLO: 0.82 - Baseline (YOLOv8n): 0.77	NR	NR	NR	- Efficacy: SRE-YOLO achieved a higher (0.82) than all compared baselines (0.70–0.77). - Efficiency: SRE-YOLO maintained the same computational efficiency (8.2 GFLOPs, 58.8 FPS) as its YOLOv8n baseline, outperforming larger models	The SRE-YOLO architecture improves the real-time detection and localization of laryngeal lesions in endoscopic images without increasing computational complexity.
Kang et al., [26]	Internal Test Set: - Accuracy: 92.78% - AUC: 0.9732 - Precision: 78.60% - Recall/Sensitivity: 76.54% - F1-score: 76.18% - Specificity: 97.61% External Test Set: - Accuracy: 85.79% - AUC: 0.9550 - Precision: 83.08% - Recall/Sensitivity: 80.98% - F1-score: 81.73% - Specificity: 95.17%	NR	NR	NR	The ILCDS (Swin-Transformer) outperformed six CNN models and the original ViT model on most metrics, especially on the external validation set. It also outperformed three expert laryngologists.	The ILCDS demonstrated superior performance compared to other AI models and human experts.
Alazwari et al., [27]	On 80% Training/20% Test Split: • Average Accuracy: 96.97% • Average Precision: 94.15% • Average Recall: 93.88% • Average F-Score: 93.88% • Average AUC Score: 95.92%	NR	NR	NR3	The proposed LCD-CMDL model outperformed all other compared models (DCNN, ResNet-50, VGG-16, etc.) in accuracy and computational speed.	The study concludes that the proposed LCD-CMDL technique is an efficient and superior method for detecting and classifying laryngeal cancer.
Du and Zu, [2]	SVM and RF machine learning models were more reliable, with smaller residual values and larger areas under the ROC curve	The study concludes that copper death-related genes “can affect the survival prognosis of laryngeal cancer patients.” WGCNA and cluster analysis were used to identify genes associated with survival prognosis.	NR	NR	SVM and RF were identified as superior to GLM and XGB for this diagnostic task	ML models (SVM and RF) can effectively leverage Copper death-related genes to build a sensitive diagnostic model for laryngeal cancer.
Wang et al., [28]	Sensitivity: 97.05% Accuracy: 99.06% Positive predictive value: 100%	NR	NR	NR	- SVM and RF showed the most robust and consistent performance improvements with synthetic data augmentation. - KNN and Gradient Boosting showed more variable performance. KNN, in particular, experienced a significant trade-off, with accuracy increasing but recall plummeting at higher augmentation levels	The study finds that CTGAN effectively augments tabular radiomics data for laryngeal cancer classification, achieving median Column Shapes and Column Pair Trends scores of 71.23% and 90.30%, respectively. This augmentation resulted in a 5% to 10% increase in accuracy across classifiers. The ideal synthetic data ratio was 100% of the original minority class, resulting in a balanced dataset.
Rajgor et al., [29]	C-index = 0.759 (95% CI 0.727–0.791)—combined model C-index = 0.655 (95% CI 0.613–0.697)—clinical-only model	Shape compacity: HR = 2.89 (95% CI 1.40–5.93), *p* = 0.004 GLZLM-GLNU: HR = 1.64 (95% CI 1.02–2.63), *p* = 0.041 5-year DSS: Upper tercile shape compacity = 51% vs. middle = 76% vs. lower = 83% (*p* = 0.032)	NR	NR	C-index: 0.759 (combined model) vs. 0.655 (clinical-only)	Two radiomic features were identified as significant prognostic indicators of disease-specific survival in advanced laryngeal cancer. Additionally, the research emphasizes that models incorporating these radiomic features performed better than those based solely on clinicopathological factors in advanced laryngeal cancer.
Rao et al., [24]	Logistic Regression: - Accuracy: 82.5% - Precision: 78.3% - Recall: 85.6% - F1-Score: 81.8% - AUC-ROC: 0.88 4-Layer Neural Network: - Accuracy: 87.2% - Precision: 83.9% - Recall: 89.7% - F1-Score: 86.7% - AUC-ROC: 0.91 ResNet-50: - Accuracy: 92.6% - Precision: 91.2% - Recall: 93.7% - F1-Score: 92.4% - AUC-ROC: 0.95	NR	NR	NR	Direct comparison of three models with increasing complexity: - Logistic Regression vs. 4-Layer NN vs. ResNet-50	Model complexity significantly impacts laryngeal cancer detection performance on CT images. ResNet-50 achieved the highest accuracy (92.6%) and best overall metrics, demonstrating superior capability in capturing fine-grained image features.
Wang et al., [30]	Best Model (DLRad_DB) Performance in Test Sets: - AUC: 0.89–0.90 - Sensitivity: 82–88% - Specificity: 79–85% Accuracy: 82–84%	Occult LNM, which is a critical prognostic factor in laryngeal cancer.	NR	Predicting occult LNM directly informs treatment decisions	The study comprehensively compared 2D DL, 3D DL, Radiomics, and two fusion strategies	The decision-based fusion model (DLRad_DB), which integrates 3D DL, 2D DL, radiomics, and clinical data, is a highly effective tool for preoperative prediction of occult LNM in LSCC.
Wang et al., [21]	Best Model (RSF) Performance on Test Set: • C-index: Glottic = 0.687; Non-Glottic = 0.657 • Integrated Brier Score (IBS) (lower is better): - Glottic (1/3/5-year): 0.116, 0.182, 0.195 - Non-Glottic (1/3/5-year): 0.130, 0.215, 0.220 • AUC: - Glottic (1/3/5-year): 0.730, 0.712, 0.703 - Non-Glottic (1/3/5-year): 0.783, 0.700, 0.682	NR	NR	NR	RSF was identified as the best-performing model for both glottic and non-glottic subtypes, outperforming CoxPH, GBM, XGBoost, and Deepsurv	The study evaluated the prognostic performance of five survival prediction algorithms across different laryngeal cancer subtypes. Among these models, the RSF algorithm demonstrated the highest performance.
Choi et al., [19]	C-Index (Concordance Index) for Survival Prediction: • DNN with Multi-classification: 0.859 ± 0.018 • DNN with Regression: 0.893 ± 0.017 • COX-PH: 0.747 ± 0.009 • RSF: 0.596 ± 0.015 • DNN with only T/N stage: 0.504 ± 0.007 Linearity Test (Ideal: slope = 1, y-intercept = 0): • DNN with Multi-classification: Slope = 1.000 ± 0.047, Y-intercept = 0.126 ± 0.762 • DNN with Regression: Slope = 0.731 ± 0.048, Y-intercept = 9.659 ± 0.964 AUC for Mortality Prediction: • DNN with Multi-classification: 0.841 ± 0.020 • DNN with Regression: 0.682 ± 0.055	Overall Survival Rates (5-year): Stage I: 84.6%, Stage II: 72.4%, Stage III: 54.2%, Stage IV: 53.9%.	Recurrence data (local: 13.9%, regional: 5.2%, distant: 3.5%)	NR	DNN models (especially multi-classification) significantly outperformed traditional statistical models (COX-PH, RSF) and a model using only T/N staging.	This study demonstrated that a multi-class DNN approach is effective for predicting survival in LSCC.
Kang et al., [31]	C-index: training (0.802 1-year, 0.789 3-year), validation (0.735 1-year, 0.746 3-year)	For Predicting Overall Survival (Nomogram): • 1-year C-index (Training): 0.802 • 1-year C-index (Validation): 0.735 • 1-year AUC (Training): 0.802 • 1-year AUC (Validation): 0.735	NR	Yes. Pathological Response (PR) to Induction Chemotherapy	The combined radiomics-clinical nomogram demonstrated superior predictive performance for OS (C-index: 0.802) compared with models using only clinical factors, such as T stage (AUC: 0.591), N stage (AUC: 0.635), or tumor volume (AUC: 0.673).	Incorporating radiomics into a nomogram, along with other clinicopathological risk factors, to estimate OS in advanced LC patients was more accurate than the clinical model alone.
Liao et al., [32]	C-index (Concordance Index): - DeepSurv: 0.71 (95% CI 0.69–0.74) - TNM Staging: 0.61 (95% CI 0.60–0.63)	The model predicts overall survival (OS). The total risk score from DeepSurv was used for risk stratification.	NR	The model identified a nonlinear association between the total risk score and treatment effectiveness. For patients with a risk score between 0.1 and 1.5, surgical treatment provided greater survival benefits than non-surgical treatment, particularly for 70.5% of stage III–IV patients.	The DeepSurv model demonstrated superior discrimination performance (C-index) compared to the traditional TNM staging system.	The DL-based model (DeepSurv) shows superior performance in predicting overall survival for LSCC patients compared to the TNM staging system.
Montenegro et al., [33]	Disease-Free Survival (DFS): 1-year DFS: 76% 3-year DFS: 66% 5-year DFS: 64% ML Models: Random Forest: 91.5% training, 93.3% validation accuracy Decision Tree: 96.6% training, 86.7% validation accuracy	LVI: 5-year DFS: LVI + 54% vs. LVI-80% (*p* < 0.05) PNI: 5-year DFS: PNI + 43% vs. PNI-78% (*p* < 0.05) Lymph Nodes: pN + 47% vs. pN0 70% (*p* < 0.05) Thyroid Infiltration: 670 mm^3^ cutoff, trend *p* = 0.08, correlation 0.7784 (*p* < 0.0001)	LVI Impact: 5-year DFS 54% (LVI+) vs. 80% (LVI−), *p* < 0.05 PNI Impact: 5-year DFS 43% (PNI+) vs. 78% (PNI−), *p* < 0.05 Cartilage Infiltration: Volume > 670 mm^3^ predicts worse prognosis		RF and DTC showed superior predictive performance. Feature Importance: Thyroid cartilage infiltration volume identified as a significant prognostic factor	The study confirmed the essential role of LVI as a prognostic indicator in advanced LSCC and highlights thyroid cartilage infiltration volume as another promising prognostic factor.
Nakajo et al., [34]	Best Model (Naïve Bayes): • Training AUC: 0.805; Accuracy: 0.727 • Testing AUC: 0.842; Accuracy: 0.813	Best Model (Random Survival Forest): • C-index (Training): 0.840 • C-index (Testing): 0.808 • The 5-year PFS rate for all patients was 57.7%.	The Naïve Bayes model predicted the recurrence.	The prediction was progression after various treatments (surgery, RT, CRT). The models were trained on data that included the treatment method.	Naïve Bayes was the best for classifying disease progression. Random Survival Forest performed best at predicting PFS, outperforming the Cox Proportional Hazards model, especially on the test set.	ML analyses using clinical and pretreatment 18F-FDG-PET-based radiomic features may help in predicting disease progression and survival in patients with laryngeal cancer.
Petruzzi et al., [3]	For 1-Year OS (J48): - Accuracy: 95% - AUC (Test): 0.886 - Precision (RD): 0.800 - Recall/Sensitivity (RD): 0.800 - F-measure (RD): 0.800 - Kappa: 0.771 (Excellent) For 3-Year OS (J48): - Accuracy: 82.5% - AUC (Test): 0.871 - Precision (RD): 0.538 - Recall/Sensitivity (RD): 0.875 - F-measure (RD): 0.667 - Kappa: 0.557 (Good)	Yes. The model predicts the oncological outcome (No Evidence of Disease (NED) vs. Relapse of Disease (RD)), including Alive with Disease or Dead of Disease, at 1 and 3 years.	Yes, the outcome “Relapse of Disease (RD)” encompasses both recurrence and persistence of disease.	NR	The J48 decision tree was compared to a Naive Bayes classifier. Results were statistically comparable (1-year accuracy: J48 95% vs. Naive Bayes 85%), demonstrating that different algorithms can produce similarly effective results.	The decision-tree ML algorithm (J48) is highly effective in predicting 1- and 3-year oncological outcomes for surgically treated laryngeal cancer patients.
Sun et al., [35]	C-index (3-year): RSF: 0.84 (training), 0.82 (validation); Cox: 0.74 (training), 0.72 (validation) C-index (5-year): RSF: 0.80 (training), 0.79 (validation); Cox: 0.75 (training), 0.72 (validation) AUC (3-year): RSF: 0.795 (training), 0.765 (validation); Cox: 0.715 (training), 0.705 (validation)	The RSF model had lower prediction errors in both the training and validation groups compared to the Cox model.	NR	NR	RSF consistently outperformed the Cox model in discrimination (C-index, AUC) and prediction error (Brier score) across both training and validation sets.	The RSF model demonstrated greater discriminative power and lower prediction error than the Cox regression model for predicting overall survival in LSCC patients.
Xu et al., [25]	Training: 98.5% accuracy, 99.9% AUC, 98.9% sensitivity, 98.2% specificity Internal Validation: 92.0% accuracy, 97.4% AUC, 91.6% sensitivity, 92.4% specificity External Validation: 86.3% accuracy, 92.6% AUC, 86.0% sensitivity, 86.6% specificity	NR	NR	NR	Densenet201 vs. Other CNNs: Superior to all other models (Alexnet 75.8%, Inception v3 78.0%, Resnet152 81.9%, Vgg19 84.1% accuracy) Densenet201 vs. Clinicians: Comparable to Clinician A (30 years, 88.1% accuracy, *p* = 0.0891) and significantly better than Clinician B (10 years, 85.3% accuracy, *p* = 0.0205).	The study confirmed the essential role of LVI as a prognostic indicator in advanced LSCC and highlights thyroid cartilage infiltration volume as another promising prognostic factor. ML holds considerable promise in augmenting traditional analysis for deriving prognostic data in laryngeal cancer.
Zhang et al., [36]	Random Survival Forest (RSF) Model: - AUC for DFS (Training): 1-year: 0.832; 3-year: 0.843; 5-year: 0.830 - AUC for DFS (Validation): 1-year: 0.739; 3-year: 0.649; 5-year: 0.640 Cox Regression Model (Nomogram): - C-index: 0.656 (95% CI 0.598, 0.694) - AUC for DFS (Training): 1-year: 0.693; 3-year: 0.655; 5-year: 0.659 - AUC for DFS (Validation): 1-year: 0.679; 3-year: 0.716; 5-year: 0.717	Hazard Ratio (HR) for DFS N Stage (higher vs. lower): 1.755 T Stage (higher vs. lower): 2.151 Pathology Grading (higher vs. lower): 1.444 Postoperative Chemoradiotherapy (Yes vs. No): 0.613 Postoperative Recovery Time (per unit increase): 0.969	Disease Progression (Recurrence/Metastasis) Data (*N* = 671) Patients with NO Progression (Non-Progress): 495 (73.7%) Patients WITH Progression (Progress) All Patients: 176 (26.3%)	NR	The RSF model showed better performance in the training cohort but similar performance in the validation cohort.	Both the RSF model and the Cox regression-based nomogram showed good predictive ability for disease-free survival in advanced LSCC. The most important prognostic variables identified were N stage, clinical stage, and postoperative chemoradiotherapy.
Li et al., [37]	Random Forest (RF): • Accuracy: 90.5% • Sensitivity: 88.2% • Specificity: 92.8% • AUC: 0.964 Convolutional Neural Network (CNN): • Accuracy: 96.1% • Sensitivity: 95.2% • Specificity: 96.9% • AUC: 0.980	NR	NR	NR	The CNN model significantly outperformed the RF model across all metrics (Accuracy, Sensitivity, Specificity, AUC).	The CNN-assisted Raman spectroscopy system achieved high accuracy, sensitivity, and specificity. These results exceeded previous research on laryngeal cancer diagnosis and conventional methods. Key features for classifying laryngeal tissues were identified, emphasizing Raman signals related to glutathione, which is known to significantly increase in laryngeal cancer.
Wang et al., [38]	OS AUC: TCGA: 3-year = 0.804, 5-year = 0.831 GSE65858: 3-year = 0.796, 5-year = 0.753 RFS/PFS AUC: TCGA: 3-year = 0.718, 5-year = 0.782 GSE65858: 3-year = 0.712, 5-year = 0.761 GSE25727: 3-year = 0.766, 5-year = 0.736 GSE27020: 3-year = 0.723, 5-year = 0.707	Cox Regression: TCGA: HR = 1.78 (95% CI 1.46–2.17), *p* < 0.001 GSE65858: HR = 1.32 (95% CI 1.06–1.74), *p* = 0.003 GSE27020: HR = 1.92 (95% CI 1.42–2.61), *p* < 0.001	RFS is significantly worse in the high-risk group	Immunotherapy response: Low-risk group: 41/55 responders (74.5%) High-risk group: 13/54 responders (24.1%)	AUC superiority: IRL classifier AUC = 0.813 vs. TNM stage AUC = 0.451 (TCGA) IRL classifier AUC = 0.805 vs. TNM stage AUC = 0.549 (GSE65858)	The IRL classifier was developed using BARX1-DT, KLHL7-DT, and LINC02154. It is designed to predict prognosis, assess immune infiltration levels, and evaluate responses to immunotherapy in patients with LSCC.
Masson et al., [39]	Training Set: AUC = 0.62, Se = 70%, Sp = 64%, *p* = 0.04 • Testing Set: Se = 80%, Sp = 67%, *p* = 0.03	NR	NR	Yes. Prediction of non-response to induction chemotherapy.	Yes. Demonstrated that harmonization improved feature generalizability. A feature that failed in the test set without harmonization performed well in training, but not in testing, even after harmonization.	Radiomic characteristics derived from CE-CT may help identify candidates for induction chemotherapy in laryngeal cancer, with fairly good sensitivity and specificity for predicting non-responsiveness. Additionally, employing statistical harmonization through ComBat and utilizing unsupervised clustering appears to enhance the predictive capabilities of features derived from a diverse multicenter environment.
Bengs et al., [40]	Reported as mean ± standard deviation over eight folds. Densenet2D • Accuracy: 0.64 ± 0.13 • Sensitivity: 0.69 ± 0.19 • Specificity: 0.62 ± 0.41 • F1-Score: 0.67 ± 0.12 Densenet2D-MS • Accuracy: 0.75 ± 0.13 • Sensitivity: 0.90 ± 0.08 • Specificity: 0.54 ± 0.24 • F1-Score: 0.76 ± 0.11 Densenet3D • Accuracy: 0.81 ± 0.09 • Sensitivity: 0.92 ± 0.12 • Specificity: 0.65 ± 0.21 • F1-Score: 0.82 ± 0.09	NR	NR	NR	Densenet3D (3D spatio-spectral) model was the most effective, outperforming both 2D-based methods across all metrics.	The study explored various DL methods for detecting laryngeal cancer in vivo using hyperspectral imaging (HSI). The study concluded that jointly learning from the spatial and spectral domains notably improves classification accuracy.
Chen et al., [41]	Radiomics Signature (Rad-score): • C-index (Training): 0.782 • C-index (Validation): 0.752 • AUC (Training): 0.783 • AUC (Validation): 0.770 Radiomics Nomogram: • C-index (Training): 0.817 • C-index (Validation): 0.913	The primary objective was to predict Overall Survival (OS). The Rad-score was a significant predictor (HR = 11.98 in training). The nomogram provided 1-year and 3-year OS probabilities.	46 patients (33.8%) developed “cancer progression” (including local relapse)	NR	The radiomics nomogram demonstrated superior predictive performance for OS compared to the AJCC staging system (C-index: 0.913 vs. 0.699 in validation, *p* = 0.019) and the clinical nomogram (*p* = 0.008 in validation).	Contrast-enhanced CT radiomics signature was independently associated with overall survival in LSCC patients. The radiomics nomogram may serve as a noninvasive, effective model to improve individualized prognostic evaluation and treatment strategies.
Smith et al., [42]	Best Model (Gradient Boosting): • Accuracy: 76.0% (95% CI 74.5–77.5) • AUC: 0.762	NR	Yes, the need for Salvage Total Laryngectomy (STL) is a direct proxy for treatment failure due to persistent or recurrent disease.	The model’s primary purpose is to predict the failure of primary non-surgical organ-preservation therapy (radiotherapy with or without chemotherapy).	The Gradient Boosting algorithm (AUC = 0.762) outperformed the LR model (AUC = 0.731).	ML shows promise as a clinical tool for identifying patients who could benefit from tailored regional care, ensuring high-quality treatment while accounting for sociodemographic factors.
Xiong et al., [43]	Binary Classification (LC + PRELCA vs. BLT + NORM): • Internal: 86.7% accuracy, 92.2% AUC, 73.1% sens, 92.2% spec • External: 89.7% accuracy, 95.3% AUC, 72.0% sens, 94.8% spec Four-Class Classification: • Internal: 74.5% accuracy • External: 77.3% accuracy	NR	NR	NR	Traditional method: 89.7% vs. 65.1% accuracy (significant) vs Human experts: Comparable to a 10–20-year expert (89.7% vs. 90.6%), better than less experienced experts	The DCNN has high sensitivity and specificity for automated detection of LC and PRELCA in laryngoscopic images, distinguishing BLT and NORM.
Zhang et al., [44]	• Independent Test Set (*n* = 33 specimens): 100% accuracy for specimen-level diagnosis. • Image-level (*n* = 80 images): AUC = 0.95; Accuracy = 90%.	NR	NR	Yes, simulated intraoperative assessment of resection margins. The AI-SRS method identified residual neoplasia at margins that appeared normal to the surgeon’s eye, directly impacting surgical outcome.	The ResNet34 model was selected and validated, and the text notes it “outperformed 4-layer MLP for the same training dataset.”	The study found that combining the DL model ResNet34 with multicolor Stimulated Raman Scattering (SRS) microscopy enables quick, accurate intraoperative diagnosis of fresh tissue samples.
Jones et al., [20]	• For Age: ANN showed significant effect (ANOVA *p* = 0.0018, log-rank *p* = 0.0025) while Kaplan–Meier did not (*p* = 0.2873) • For N Stage: All methods showed significant effects (*p* = 0.0004–0.0008)	Survival Analysis No significant differences between the three methods: ANOVA: *p* = 0.8965 Log-rank test: *p* = 0.8110	NR	NR	ANN was significantly more sensitive than Cox’s model: • Detected survival differences for age and N stage that Cox’s model missed • Produced enhanced dichotomy between risk groups • Showed increasing separation over time (χ^2^ for trend *p* = 0.0025)	The Kaplan–Meier plot showed no significant differences in overall survival rates. While the network generated results qualitatively similar to those from the Cox model, it showed greater sensitivity to variations in survival trends by age and N stage. The study suggests that neural networks are adept at making predictions in systems characterized by intricate interactions among variables and non-linear relationships.

AJCC: American Joint Committee on Cancer, ANN: Artificial Neural Network, AUC: Area Under the Curve, BLT: Benign Laryngeal Tumor, CE-CT: Contrast-Enhanced Computed Tomography, CI: Confidence Interval, CNN: Convolutional Neural Network, DCNN: Deep CNN, COX-PH: Cox Proportional Hazards, CRT: Chemoradiotherapy, CSS: Cancer-Specific Survival, CTGAN: Conditional Tabular Generative Adversarial Network, DFS: Disease-Free Survival, DNN: Deep Neural Network, GBC: Gradient Boosting Classifier, GLZLM-GLNU: Gray Level Zone Length Matrix Gray Level Non-Uniformity, HR: Hazard Ratio, HSI: Hyperspectral Imaging, ILCDS: Intelligent Laryngeal Cancer Detection System, LC: Laryngeal Cancer, LNM: Lymph Node Metastasis, LOOCV: Leave-One-Out Cross-Validation, LR: Logistic Regression, LSCC: Laryngeal Squamous Cell Carcinoma, LVI: Lympho-Vascular Invasion, NED: No Evidence of Disease, NORM: Normal, NR: Not Reported, OS: Overall Survival, PFS: Progression-Free Survival, PNI: Perineural Invasion, PRELCA: Precancerous Laryngeal Lesion, RD: Relapse of Disease, RF: Random Forest, RFS: Recurrence-Free Survival, ROC: Receiver Operating Characteristic, RSF: Random Survival Forest, RT: Radiotherapy, STL: Salvage Total Laryngectomy, SVM: Support Vector Machine, TNM: Tumor, Node, Metastasis, XGB: XGBoost, RFC: Random Forest Classifier, RF: Random Forest, ML: Machine Learning, DL: Deep Learning.

## Data Availability

Data presented in this study are available upon reasonable request from the corresponding author.

## Abbreviations

The following abbreviations are used in this manuscript:

AI	Artificial intelligence
DL	Deep Learning
GEO	Gene Expression Omnibus
HNC	Head and neck cancer
LC	Laryngeal cancer
LSCC	Laryngeal squamous cell carcinoma
ML	Machine learning
RSF	Random survival forest
TNM	Tumor, node, metastasis
